# ELLIPSE Resilience Enhancement (ERE): A Multidisciplinary Approach of Suicide Prevention Training for Higher Education Students

**DOI:** 10.1192/j.eurpsy.2026.11969

**Published:** 2026-07-31

**Authors:** J. Gustavsson, A. De La Torre-Luque, J. M. Andreu-Rodriguez, A. Baran, L. Brau, B. Chechotka, J. Cuny, M. E. De la Peña-Fernandez, O. Gawlik-Kotelnicka, A. Gmitrowicz, J. Grzejszczak, N. Lagunas, S. Lesinskienė, E. Łojewska, V. Lönnfjord, L. Meiländer, T. Milewicz, M. P. Munuera, K. Pociūtė, L. Rakhman, K. Rosa, B. Schneider, D. Strzelecki, M. Wik, A. Zalewska-Janowska

**Affiliations:** 1 Karlstad University, Karlstad, Sweden; 2 Complutense University of Madrid, Madrid, Spain; 3 Euorpean Student’s Union, Brussels, Belgium; 4 Lviv District Clinical Psychiatric Hospital, Lvov, Ukraine; 5 Medical University of Lodz, Lodz, Poland; 6 Vilnius University, Vilnius, Lithuania; 7 LVR-Klinik Köln, Köln, Germany; 8 Jagiellonian University in Krakow, Krakow, Poland; 9 Danyla Halytsky Lviv National Medical University, Lvov, Ukraine

## Abstract

**Introduction:**

Suicide is a public health issue with immense personal and economic costs. While education is highlighted as a key preventive measure, a critical gap exists in higher education, where few programs offer comprehensive suicide prevention training, leaving students and teachers underprepared. The ELLIPSE Resilience Enhancement (ERE) project addresses this deficit by creating a scalable, sustainable, and innovative curriculum to improve knowledge and skills in academic communities. Its primary objective is to strengthen academic resilience by developing a comprehensive suicide prevention curriculum, conducting a large-scale needs assessment, developing innovative materials, and advocating for the integration of suicide prevention into university curricula and national policies. The project also supports the cooperation with Ukrainian universities, promoting inclusivity and innovative teaching practices.

**Objectives:**

To present and describe the ERE project.

**Methods:**

ERE is organized into five interdependent work packages (Figure 1): WP1. Project Management: Oversees the project’s successful implementation and monitoring. WP2. Needs Assessment and Challenges Inventory: Conducts international surveys to gather data from over 1200 participants and identifies stakeholder needs. WP3. Course Development and Innovation: Develops an online e-learning course, risk scenarios, and the 12-Step Safety Plan for Academia digital tool. WP4. European Summer School of Suicidology and Alumni Network: Tests the educational resources developed in WP3 and establishes an alumni network for ongoing knowledge exchange. WP5. Evaluation and Impact Assessment: Evaluates the program’s impact and develops a comprehensive dissemination strategy.

The project is led by Karlstads University (Sweden). The consortium includes a diverse partnership of academic institutions and organizations from six European countries (Poland, Spain, Lithuania, Ukraine, Belgium and Germany).

**Results:**

Expected results include a comprehensive needs assessment, a tailored online curriculum, a robust evaluation framework, and a policy document advocating for curriculum integration. The project will deliver the European Summer School of Suicidology and the online course will be available as an open educational resource.

**Image 1:**
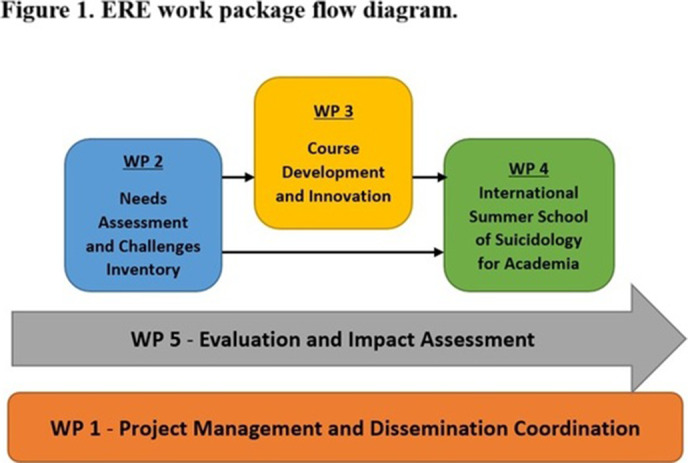
Image 1: Long description.

**Conclusions:**

The ERE project addresses a pressing need for suicide prevention training in higher education by developing and disseminating a multidisciplinary, evidence-based curriculum. The project’s outcomes will be integrated into institutional policies and national suicide prevention strategies. By leveraging the expertise of its partners and providing accessible digital tools, the project ensures long-term sustainability and a lasting positive impact on student mental health and well-being. This investment is highly cost-effective, with a potential return of 31 EUR for every 1 EUR invested.

**Disclosure of Interest:**

None Declared

## Long descriptions

### Image 1: Long description

The flowchart is organized into two main sections.

Top Section: Three colored boxes arranged horizontally with arrows indicating workflow.

* Left: A blue box labeled W P 2 Needs Assessment and Challenges Inventory. Two arrows point right from this box.

* Center: A yellow box labeled W P 3 Course Development and Innovation. One arrow from W P 2 enters this box, and one arrow exits to the right.

* Right: A green box labeled W P 4 International Summer School of Suicidology for Academia. It receives arrows from both W P 2 and W P 3.

Bottom Section: Two wide horizontal bars spanning the width of the diagram.

* Upper bar: A grey arrow pointing right labeled W P 5 - Evaluation and Impact Assessment.

* Lower bar: An orange rounded rectangle labeled W P 1 - Project Management and Dissemination Coordination.

Navigate back to Image 1:.

